# Characterisation and radioimmunotherapy of L19-SIP, an anti-angiogenic antibody against the extra domain B of fibronectin, in colorectal tumour models

**DOI:** 10.1038/sj.bjc.6603806

**Published:** 2007-05-22

**Authors:** E El-Emir, J L J Dearling, A Huhalov, M P Robson, G Boxer, D Neri, G A M S van Dongen, E Trachsel, R H J Begent, R B Pedley

**Affiliations:** 1 Cancer Research UK Targeting and Imaging Group, Department of Oncology, Royal Free and University College Medical School (UCL), Hampstead Campus, Rowland Hill Street, London NW3 2PF, UK; 2 Institute of Pharmaceutical Sciences, Department of Chemistry and Applied Biosciences, Swiss Federal Institute of Technology Zurich, Wolfgang-Pauli-Str. 10, ETH Hoenggerberg, HCI G396, Zurich CH-8093, Switzerland; 3 Department of Otolaryngology/Head and Neck Surgery, VU University Medical Centre, De Boelelaan 1117, Amsterdam 1081 HV, The Netherlands

**Keywords:** L19-SIP, radioimmunotherapy, ED-B, vasculature, tumour

## Abstract

Angiogenesis is a characteristic feature of tumours and other disorders. The human monoclonal antibody L19- SIP targets the extra domain B of fibronectin, a marker of angiogenesis expressed in a range of tumours. The aim of this study was to investigate whole body distribution, tumour localisation and the potential of radioimmunotherapy with the L19-small immunoprotein (SIP) in colorectal tumours. Two colorectal tumour models with highly different morphologies, the SW1222 and LS174T xenografts, were used in this study. Localisation and retention of the L19-SIP antibody at tumour vessels was demonstrated using immunohistochemistry and Cy3-labelled L19-SIP. Whole body biodistribution studies in both tumour models were carried out with ^125^I-labelled L19-SIP. Finally, ^131^I-labelled antibody was used to investigate the potential of radioimmunotherapy in SW1222 tumours. Using immunohistochemistry, we confirmed extra domain B expression in the tumour vasculature. Immunofluorescence demonstrated localisation and retention of injected Cy3-labelled L19-SIP at the abluminal side of tumour vessels. Biodistribution studies using a ^125^I-labelled antibody showed selective tumour uptake in both models. Higher recorded values for localisation were found in the SW1222 tumours than in the LS174T (7.9 *vs* 6.6 %ID g^−1^), with comparable blood clearance for both models. Based on these results, a radioimmunotherapy study was performed in the SW1222 xenograft using ^131^I-Labelled L19-SIP (55.5 MBq), which showed selective tumour uptake, tumour growth inhibition and improved survival. Radio- and fluorescence-labelled L19-SIP showed selective localisation and retention at vessels of two colorectal xenografts. Furthermore, ^131^I-L19-SIP shows potential as a novel treatment of colorectal tumours, and provides the foundation to investigate combined therapies in the same tumour models.

Radioimmunotherapy (RIT), the targeting of radiation to the tumour using monoclonal antibodies against tumour-associated antigens, has been used as a form of cancer treatment in both animal xenograft models as well as in humans (Pedley *et al*, 2001; Koppe *et al*, 2005). This form of therapy has the advantage of selectively targeting the tumour while reducing systemic toxicity. Solid tumours possess a heterogeneous pathophysiology and, therefore, the effectiveness of RIT depends on a range of factors including distribution of antibody uptake within the whole tumour mass and tumour vascularity.

Angiogenesis, the development of new blood vessels from pre-existing vessels, is a common feature of solid tumours and other diseases such as blinding ocular disorders and rheumatoid arthritis. Angiogenic processes are regulated by a number of cell surface receptors and extracellular adhesion molecules (Ebbinghaus *et al*, 2004). The ability to selectively target angiogenic molecules (markers) is useful for the delivery of therapeutic agents to the tumour. One potential marker for neovasculature is the extra domain B (ED-B) of fibronectin (FN).

Fibronectins are high-molecular-weight-adhesive glycoproteins abundantly expressed *in vivo* in the extracellular matrix and in body fluid (Kaspar *et al*, 2006). These play an important role in a number of biological phenomena such as maintenance of normal cell morphology, cell migration, haemostasis and thrombosis, wound healing, and oncogenic transformation (Castellani *et al*, 1994). Alternative splicing in the primary transcript results in multiple FN transcripts of the single copy gene. Three FN isoforms are expressed in tumour tissue and transformed cells, these include IIICS, extra domain A and ED-B (Ebbinghaus *et al*, 2004; Kaspar *et al*, 2006).

Extra domain-B domain is a complete type III repeat composed of a sequence of 91 amino acids, coded by a single exon and is the most conserved region of FN. It displays 100% homology in all mammalian species (Zardi *et al*, 1987). It is usually absent in both plasma and tissue FN of adults, except in regenerating tissues, such as some vessels in the endometrium. However, it is overexpressed during active tissue remodelling, for example, angiogenesis in tumours, wound healing and embryogenesis (Ebbinghaus *et al*, 2004; Kaspar *et al*, 2006). It has been shown to accumulate specifically around neovasculature in studies of many different tumour types, making it a good marker for angiogenesis (Carnemolla *et al*, 1989; Kaczmarek *et al*, 1994; Neri *et al*, 1997; Kosmehl *et al*, 1999; Tarli *et al*, 1999; Viti *et al*, 1999; Demartis *et al*, 2001; Castellani *et al*, 2002; Birchler *et al*, 2003; Ebbinghaus *et al*, 2004).

Human antibodies against the ED-B domain of FN, termed ‘L19’, have been isolated using different technologies (Pini *et al*, 1998; Borsi *et al*, 2002; Alessi *et al*, 2004). These include a complete human IgG, dimeric scFv ((scFv)_2_) and a small immunoprotein (SIP). The L19-SIP is an 80 kDa fragment composed of two scFv fused to the human ∈_s2_–CH4 domain to provide a covalent stabilisation of the dimer (Li *et al*, 1997; Borsi *et al*, 2002).

Several fusion proteins based on the L19 antibody have been produced and characterised (Birchler *et al*, 1999; Nilsson *et al*, 2001; Halin *et al*, 2002; Halin *et al*, 2003; Ebbinghaus *et al*, 2005; Fabbrini *et al*, 2006). Furthermore, several radiolabelled derivatives of the L19 antibody have been studied in biodistribution experiments (Borsi *et al*, 2002) as well as being evaluated in clinical radioimmunscintigraphy studies (Santimaria *et al*, 2003). Biodistribution experiments and RIT studies with the L19-SIP antibody have been carried out in tumour-bearing mice and rats, and have demonstrated selective localisation in different tumour models, for example, human melanomas, mouse embryonal teratocarcinomas, human head and neck squamous cell carcinomas, and rat gliomas (Borsi *et al*, 2002; Berndorff *et al*, 2005; Spaeth *et al*, 2006; Tijink *et al*, 2006). Finally, the therapeutic potential of ^131^I-L19-SIP is currently being investigated in clinical trials in Italy and Switzerland (Kaspar *et al*, 2006).

In the present study, we have investigated for the first time, the distribution of the L19-SIP antibody in two human colorectal tumour models (SW1222 and LS174T), using both radio- and fluorescence-labelled antibody. We demonstrate that the radiolabelled antibody localises selectively in both tumour models. Furthermore, by employing high-resolution multi-fluorescence microscopy, we have been able to show that the antibody localisation is specific to the abluminal surface of the vessel. Finally, we show that RIT with ^131^I-L19-SIP causes tumour growth retardation and prolongs survival in preliminary therapy studies.

## MATERIALS AND METHODS

### Antibody

The L19-SIP (Mw 80 kDa), which targets the ED-B domain of FN consists of a fusion between two scFv and a CH4 domain of the human IgE, which mediates the homodimerisation of recombinant antibody (Borsi *et al*, 2002).

### Xenografts

The human colorectal adenocarcinoma cell lines SW1222 and LS174T were used to develop subcutaneous xenograft models in the flanks of female nude mice (Pedley *et al*, 2001). SW1222 tumours are well differentiated and highly vascularised in comparison to the less differentiated and more poorly vascularised LS174T tumours (Pedley *et al*, 2002), and both represent morphologies frequently observed in the clinic.

Passaging was by subcutaneous implantation of small tumour pieces (∼1 mm^3^). Biodistribution experiments were carried out when tumours reached approximately 1 cm^3^. For therapy experiments, mice with palpable tumours of approximately 0.1 cm^3^ were used. All animal experiments were in compliance with the UK Co-ordinating Committee on Cancer Research (UKCCCR) guidelines for the Welfare of Animals in Experimental Neoplasia.

For immunohistochemistry and immunofluorescence staining, tumours were isolated, snap frozen in liquid nitrogen and stored at −80C.

### ED-B antigen expression

Immunohistochemistry was performed on 10 *μ*m thick cryostat sections of both SW1222 and LS174T tumours after fixing in acetone for 10 min at room temperature. Following blocking of endogenous peroxidase with 0.3% of H_2_O_2_/methanol for 15 min at room temperature, sections were first incubated in 3% normal goat serum/phosphase-buffered saline (PBS) for 30 min, followed by a1 h incubation with 2 *μ*g ml^−1^ L19-SIP antibody at room temperature. After washing with PBS, the sections were then incubated with a horseradish peroxidase conjugated goat anti-human antibody (DAKO UK Ltd, Ely, UK) for 1 h at room temperature, rinsed in PBS, and then incubated with the avidin–biotin complex (Vector Laboratories Ltd, Orton Southgate, UK) following the manufacturer's instructions. Sections were developed with 3,3-diaminobenzidine (Sigma-Aldrich Company Ltd, Gillingham, UK) and counterstained with haematoxylin. Finally, sections were mounted in dibutyl phthalate xylene and visualised under a light microscope.

### Fluorescence-labelled antibody studies

#### Fluorescence labelling

The L19-SIP antibody was labelled with Cy3-NHS (Amersham Pharmacia, Little Chalfont, UK) according to the manufacturer's instructions. The final antibody to dye ratio was 1 : 5. A total of 100 *μ*g of labelled antibody was injected intravenously into the tail of the mouse 6 h before killing. Tumours were then excised, snap frozen in isopentane (cooled in liquid nitrogen), and stored at −80°C until sectioned.

#### Tumour parameters

To relate Cy3-L19-SIP antibody distribution to tumour morphology/pathophysiology, the following parameters were studied by multi-fluorescence microscopy:

1. *Perfusion*: the *in vivo* DNA-binding dye Hoechst 33342 (10 mg kg^−1^) was injected intravenously 1 min before the mice were killed.
2. *Blood vessels*: an anti-CD31 antibody was used to stain for blood vessel distribution, and the relevant immunohistochemical staining procedures were performed (see below).
3. *Nuclear staining*: DAPI (4,6 diamidino-2-phenylindole) was used as a nuclear marker. This produces a blue fluorescence when bound to DNA.

#### Multi-fluorescence microscopy

Sections (10 *μ*m) were first fixed in acetone for 10 min at room temperature, and then incubated with a 1 : 2 dilution of unconjugated anti-CD31 rat anti-mouse antibody (a kind gift from Professor A Mantovani) for 1 h at room temperature. After rinsing in PBS, sections were incubated with a 1 : 200 dilution of an Alexa Fluor 488 goat anti-rat antibody (Invitrogen Ltd, Paisley, UK) for 1 h at room temperature. After further rinsing with PBS, sections were mounted in either mounting medium containing DAPI (Vector Laboratories) or in PBS and viewed using an Axioskop 2 microscope (Carl Zeiss Ltd, Welwyn Garden City, UK), fitted with a computer-controlled motorised stage. Images were captured by an AxioCam digital colour camera using KS300 image analysis software (Zeiss, UK) (Pedley *et al*, 2002; El-Emir *et al*, 2005). Briefly, injected Hoechst 33342 and DAPI staining were visualised under a UV filter. Injected Cy3-L19-SIP and the CD31 signal were visualised under rhodamine and FITC filters, respectively. Both composite tiled images, consisting of a large number of individual fields, as well as high-resolution single images, for three different fluorophores (stained for three different parameters), were generated. Finally, the fluorescence images were then co-registered using Adobe Photoshop software, resulting in a new multi-channel image showing the inter-relationship between the overlapping fluorescently labelled structures.

### Radiolabelled antibody studies

#### ^125^I-labelling

L19-SIP was labelled according to the IODO-GEN method as described by Visser *et al* (2001). Briefly, to an IODO-GEN-coated glass tube, the following was added: 50 *μ*l 0.5 M phosphate buffer, pH 7.4; 250 *μ*g L19-SIP (0.5 mg ml^−1^) in 0.1 M phosphate buffer, pH 6.8; 50.18 MBq ^125^I (Amersham Biosciences). The reaction was stopped after 4 min by the addition of 100 *μ*l of ascorbic acid (25 mg ml^−1^, pH 5). The solutions from two separate labellings were combined and purified on a PD-10 column and eluted with 0.9% NaCl/ascorbic acid (5 mg ml^−1^, pH 5.0). For the PD-10 column, the first 2.5 ml (1 ml sample volume and the first 1.5 ml) were discarded and the radiolabelled L19-SIP was collected in the next 2 ml.

#### ^131^I-labelling

Preparation of ^131^I-L19-SIP for therapy was performed according to the ‘IODO-GEN-coated MAb method’ (Visser *et al*, 2001; Tijink *et al*, 2006). Briefly, ^131^I (444 MBq) was added to a glass vial in 1 ml NaOH (1 mmol l^−1^), after which 10 *μ*l of ascorbic acid (1.4 mg ml^−1^) was added. After 5 min, 400 *μ*l of phosphate buffer (0.5 M, pH 7.2) was added, followed by 3 ml L19-SIP (approximately 1.5 mg) and 35 *μ*l IODO-GEN solution (1 mg ml^−1^). After 3 min, the reaction was stopped by adding 100 *μ*l ascorbic acid (25 mg ml^−1^) followed after 5 min by the addition of 50 *μ*l human serum albumin (20%). The solution was then purified on a PD-10 column and eluted with 0.9% NaCl/ascorbic acid (5 mg ml^−1^, pH 5.0). The initial 2.7 ml eluate (2.2 ml sample volume and the first 0.5 ml) was discarded and the radiolabelled L19-SIP was collected in the next 3.0 ml.

#### Analyses

Thin layer chromatography (TLC) was used to assess radiopharmaceutical purity (silica gel Si_60_ stationary phase and citrate buffer (20 mM, pH 5.0) mobile phase).

The immunoreactivity of the radiolabelled protein was assessed on a 200 *μ*l volume ED-B sepharose column saturated with 0.25% BSA in PBS. An aliquot of radiolabelled antibody was applied to the column, which was then rinsed with 1.5 ml of 0.25% BSA in PBS to remove non-specifically bound antibodies. Bound material was eluted using 1.5 ml of 0.1 M TEA, pH 11. The radioactivity of the bound and unbound material was counted, using a *γ*-counter, and the immunoreactive percentage was calculated.

#### Biodistribution of ^125^I-L19-SIP

Biodistribution experiments with the ^125^I-L19-SIP were carried out in both SW1222 and LS174T xenograft-bearing mice. Mice were injected in the tail vein with 8 *μ*g (0.26 MBq) of radiolabelled antibody and dissected at 1, 3, 6 or 24 h after injection. For each time point, four mice were killed and the following organs were excised, weighed and counted in a *γ*-counter (Cobra Multi Gamma Model 5010-Packard, UK): blood, tumour, liver, spleen, kidney, intestine, heart, lung, brain, muscle, stomach and tail. The radioactive content of the different organs was expressed as the percentage of injected dose per gram of tissue (%ID g^−1^). Biodistribution experiments were analysed using Microsoft office Excel 2003.

#### Therapy

Therapy experiments with ^131^I-L19-SIP were performed on SW1222 xenograft-bearing mice. To confirm that the ^131^I-labelled L19-SIP was selectively localizing to the tumour, and to compare localisation with ^125^I-labelled L19-SIP, a biodistribution study was first performed with the former. Each mouse was injected in the tail vein with 1.6 *μ*g (0.75 MBq) of radiolabelled antibody, and groups of four mice killed at 3, 24 and 72 h after injection. Later time points (i.e., 24 and 72 h) were included to investigate prolonged antibody retention, which is important for RIT efficacy. The following organs were excised, weighed and counted in a *γ*-counter: blood, tumour, liver, spleen, kidney, lung, colon, muscle, brain and stomach. The radioactive content of the different organs was expressed as %ID g^−1^.

For the RIT experiments, a group of four mice was injected, intraperitonially, with 117.5 *μ*g (55.5 MBq) ^131^I-L19-SIP per mouse. A second group of six mice were used as untreated controls.

During treatment, tumours were measured twice weekly for a period of approximately 140 days, and tumour volume was estimated as the measurement of length × width × height/2 (Pedley *et al*, 2001). As well as observing general condition (e.g., respiratory distress, abnormal behaviour and no food/fluid intake), toxicity was assessed by using body weight measured every 3–4 days, as a surrogate marker. Mice were killed when tumours reached approximately 1.5 cm^3^ in volume. Therapy experiments were analysed using Microsoft office Excel 2003 and SPSS software. Survival was calculated using Kaplan–Meier analysis and showed significant correlation using the log-rank test (*P*=0.0034). Suppression of tumour growth was analysed using a non-parametric test (Mann–Whitney *U*-test). This showed a significant difference in tumour size between untreated and therapy groups at day 13 (*P*=0.038), which was sustained up until day 25 (*P*=0.011).

## RESULTS

### Antigen expression

**Figure 1:**
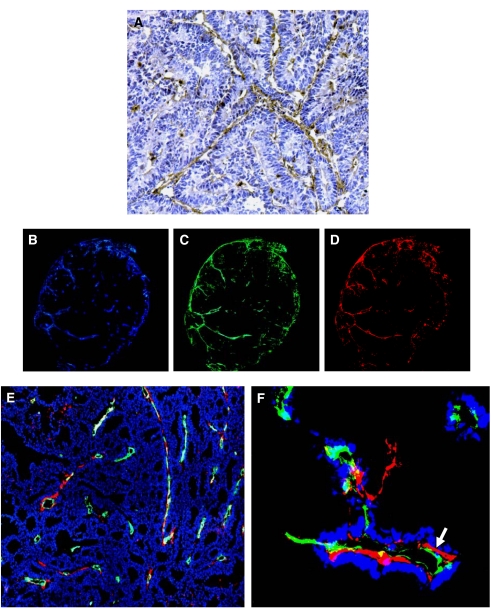
Immunohistochemical and immunofluorescence staining with L19-SIP antibody in SW1222 and LS174T xenograft-bearing mice. (**A**) Immunohistochemical staining of SW1222 xenograft lines with L19-SIP antibody. (**B–D**) Multiple digital fluorescence images of an LS174T tumour injected with Cy3-labelled L19-SIP antibody for 6 h demonstrating (**B**) perfusion, (**C**) blood vessel staining and (**D**) L19-SIP in the same tumour section. (**E**) Triple fluorescence staining of CD31 (green), L19-SIP (red) and DAPI (blue) in LS174T tumours. (**F**) High-power image of CD31 (green), L19-SIP (red) and Hoechst (blue) staining in LS174T (arrow indicating abluminal localisation of the L19-SIP antibody). All images are at × 20 magnification except (**F**) which is at × 40 magnification.

Before distribution studies with fluorescence-labelled L19-SIP, tumours were investigated for ED-B expression. Immunohistochemistry with the L19-SIP antibody confirmed that ED-B expression was associated with the majority of blood vessels as demonstrated in the SW1222 tumour model (Figure 1A). This was observed throughout the whole of the tumour.

### Distribution of fluorescence labelled L19-SIP

Triple immunofluorescence staining of vessels (CD31), L19-SIP and perfusion (Hoechst 33342) over whole tumour sections, demonstrated that the antibody was associated with blood vessels in both peripheral and central regions of the tumour in the LS174T xenograft model (Figure 1B–D). However, co-registration of fluorescence staining for blood vessels, L19-SIP and cell nuclei (DAPI), revealed that not all CD31-stained vessels were L19-positive (Figure 1E), indicating that ED-B was not associated with all tumour vessels. Furthermore, co-registration of fluorescence staining for CD31, L19-SIP and perfusion revealed that the L19-SIP had localised to the abluminal surface of the vessels, confirming that expression of EDB was in the extracellular matrix (i.e., stroma) of the tumour (Figure 1F).

### Radiolabelled L19-SIP

As mentioned in the Materials and Methods section, L19-SIP antibody was labelled following the IODO-GEN method with both ^125^I and ^131^I. Preliminary studies showed that the protein could be labelled using the methods described above and a plateau of labelling was found at 4 min. Antigen binding of the labelled protein was found to be 91.7% for ^125^I-L19-SIP and 83.7% for ^131^I-L19-SIP. In addition, stability of the ^125^I-L19-SIP was assessed by TLC, and was found to be 99.2% after 5 days post-labelling.

### Biodistribution of ^125^I- and ^131^I-labelled L19-SIP

**Figure 2:**
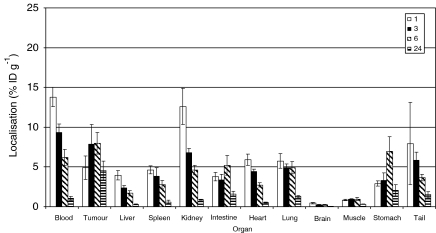
Biodistribution of intravenously injected ^125^I-L19-SIP in SW1222 xenograft-bearing mice at 1, 3, 6 and 24 h after injection. The mean±s.d. of four different mice are shown.

**Figure 3:**
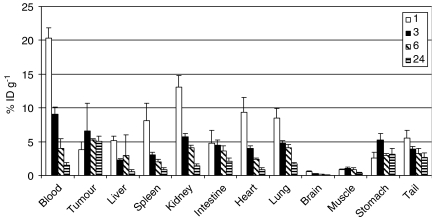
Biodistribution of intravenously injected ^125^I-L19-SIP in LS174T xenograft-bearing mice at 1, 3, 6 and 24 h after injection. The mean±s.d. of four different mice are shown.

To compare localisation of the L19-SIP antibody in the two colorectal xenograft models, biodistribution studies were carried out using ^125^I-labelled antibody (Figures 2 and 3). In SW1222 tumours, the %ID g^−1^ reached a maximum of 7.9 (±1.3) at 6 h after injection. In comparison, this reached a maximum of 6.6 (±4) at 3 h in the LS174T tumours. At 24 h post-injection, the %ID g^−1^ was 4.5 (±1.2) and 5 (±0.6) for SW1222 and LS174T, respectively.

**Figure 4:**
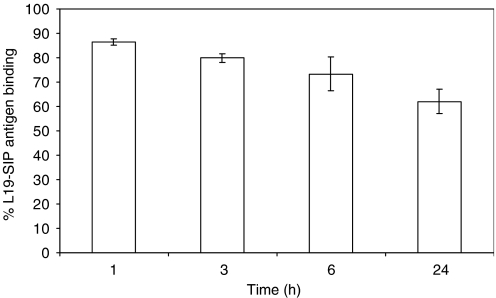
Blood stability data. Antigen binding of ^125^I-L19-SIP in blood of LS174T tumour-bearing mice at 1, 3, 6 and 24 h post-injection. The mean±s.d. of four different mice are shown. Binding of the labelled protein before injection was 84.11%.

Blood stability studies showed that at 1 h 86.45±1.39% of activity was associated with active protein (binding to antigen), reducing to 62.01±4.9% after 24 h, in LS174T-bearing mice (Figure 4). This was confirmed after the bloods from the different time points had been run (Figure 4).

Tumour to blood ratios for the two tumour models are shown in Table 1. Initial blood clearance was faster in the SW1222 line than in the LS174T, but by 6 h post-injection, the blood clearance for both tumour models was comparable, with tumour to blood ratios of approximately 1.28. However, at 24 h, there was a higher tumour to blood ratio for the SW1222, which reached 4.22 in comparison to 3.37 for the LS174T. This led to a higher area under curve for the SW1222, measuring 151.6 %ID g^−1^ h^−1^ *vs* 122.6 for the LS174T tumours.

**Figure 5:**
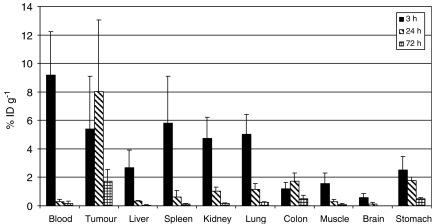
Biodistribution of intravenously injected ^131^I-L19-SIP in SW1222 xenograft-bearing mice at 3, 24 and 72 h after injection. The mean±s.d. of four different mice are shown.

On the basis of these results, the SW1222 colorectal model was selected for a RIT study. A biodistribution study with the ^131^I-labelled L19-SIP was, therefore, carried out at 3, 24 and 72 h post-injection, to confirm the tumour specificity seen with ^125^I-labelled L19-SIP and to investigate long-term retention of antibody for RIT. Antibody distribution in tumour, blood and normal tissues is shown in Figure 5. Uptake values in the tumour at 3 and 24 h were 5.4 (±3.6) and 8 (±5) %ID g^−1^, respectively. By 72 h, 1.7 (±0.8) %ID g^−1^ was still retained in the tumour.

In addition, there was an increase in tumour to blood ratio of antibody over time, rising from 0.58 at 3 h to a maximum of 28 at 24 h. Although this ratio decreased by 72 h, it remained relatively high at 9.3 (Table 2).

### Radioimmunotherapy

**Figure 6:**
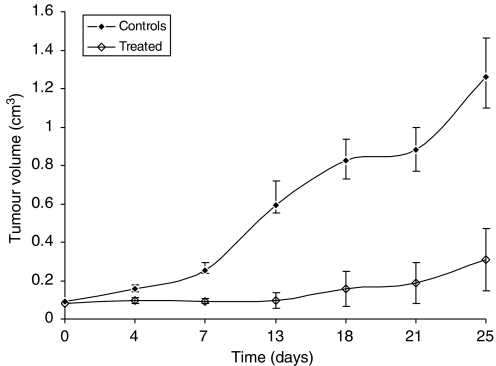
Tumour volumes of control and treated SW1222 xenografts following a single injection of 55.5 MBq of ^131^I-L19-SIP. The mean±s.e.m. of six mice for the control and four mice for the treated groups are shown.

**Figure 7:**
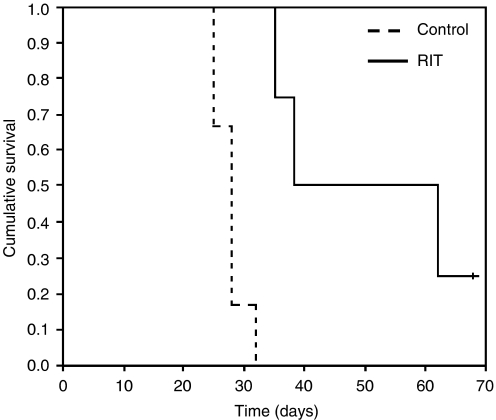
Survival of SW1222 xenograft-bearing nude mice after a single injection of 55.5 MBq of ^131^I-L19-SIP.

Radioimmunotherapy with ^131^I-L19-SIP produced a significant effect on tumour growth and survival in SW1222 xenograft-bearing mice. Treatment with a single dose of 55.5 MBq mouse^−1^ inhibited subsequent tumour growth for a mean of approximately 14 days (Figure 6) in comparison to control untreated mice, which showed a steady increase in tumour growth throughout the experiment. With the exception of one mouse, all tumours in the therapy group subsequently re-grew, although at a slower rate to that of controls. In addition to arrested tumour growth, RIT also resulted in a prolongation of survival (Figure 7). While all mice in the control group had been culled by day 32, this was extended to 68 days for three of the treated mice. However, one out of four treated mice is apparently cured at 193 days post-injection.

**Figure 8:**
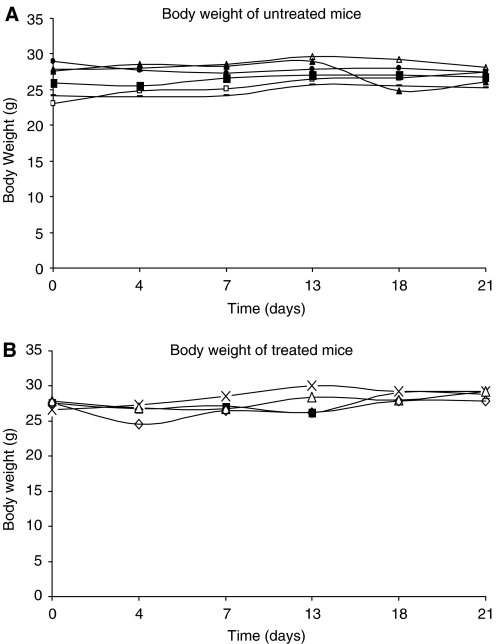
Individual body weights of (**A**) control and (**B**) treated mice bearing SW1222 xenografts, after a single injection of 55.5 MBq of ^131^I-L19-SIP.

Toxicity was assessed by regular weighing, and individual mouse weights are shown in Figure 8. In the treated group, mean body weight reached a nadir of 4% of the starting weight at day 4 post-therapy. Initial weight was regained at day 7, and subsequently rose throughout the experiment. In comparison, control mice showed a mean weight loss of 1% at day 4 with a recovery exceeding initial values by day 7.

## DISCUSSION

In the present study, the distribution of the L19-SIP antibody in both SW1222 and LS174T colorectal tumour models has been investigated using both radio- and fluorescence-labelled antibodies. Furthermore, we have examined the potential of RIT against tumour vasculature in colorectal cancer, using ^131^I in the SW1222 tumour model.

Using immunohistochemistry, the presence of ED-B was demonstrated around the vasculature in both tumour models (Figure 1A). This is supported by other studies in different tumour types (Zardi *et al*, 1987; Carnemolla *et al*, 1989; Castellani *et al*, 1994; Kaczmarek *et al*, 1994; Neri *et al*, 1997; D’Ovidio *et al*, 1998; Kosmehl *et al*, 1999; Tarli *et al*, 1999; Viti *et al*, 1999; Ebbinghaus *et al*, 2004).

We subsequently demonstrated, by multi-fluorescence microscopy, that fluorescence-labelled L19-SIP, when injected intravenously, localised specifically to tumour vessels at 6 h post-injection (Figure 1B–E). This distribution pattern was still retained at 24 h post-injection (data not shown). Fabbrini *et al* (2006) also demonstrated perivascular localisation of the L19-SIP in F9 teratocarcinoma-bearing mice. However, using high magnification, we could clearly show for the first time that the antibody is retained on the abluminal surface of the tumour blood vessels (Figure 1F).

We also found that not all vessels stained positive for L19-SIP (Figure 1E), suggesting that ED-B expression may only be related to the neovasculature (i.e., immature newly formed vessels) of our two colorectal tumour models. To clarify this, we are currently using fluorescence microscopy and quantitative analysis to investigate whether a correlation pattern exists between EDB expression/L19-SIP localisation and blood vessel maturation in both SW1222 and LS174T tumour models. Specifically, we are looking at different vessel maturation markers, including those of the pericyte/mural cells as well as basement membrane markers, in different sized tumours. Preliminary data show that the majority of blood vessels within a tumour stain for both CD31 and L19-SIP regardless of tumour size, in comparison to control non-tumoural tissue which shows no L19-SIP staining around the blood vessels. These finding will be crucial when designing combined therapies with the L19-SIP antibody.

Having confirmed ED-B expression in the two colorectal xenografts, biodistribution experiments were performed. ^125^I-labelled L19-SIP antibody demonstrated good tumour uptake and localisation as early as 1 h post-injection in both models (Figures 2 and 3). However, we observed higher localisation of the L19-SIP antibody in the SW1222 tumours, which reached a maximum of 7.9 %ID g^−1^ at 6 h. In comparison, the LS174T tumours reached maximum of 6.6 %ID g^−1^ at 3 h after injection. This difference in the L19-SIP antibody distribution may reflect the state of vascularisation within these tumours: the well-differentiated SW1222 tumours are highly vascularised in comparison to the less-differentiated LS174T tumours, which are poorly vascularised and therefore less accessible to the protein.

Blood clearance of ^125^I-L19-SIP antibody in both tumour models was comparable, with tumour to blood ratios of approximately 1.28 for both tumours at 6 h (Table 1). Borsi *et al* (2002), also demonstrated maximum localisation of the ^125^I-L19-SIP, 6.1 %ID g^−1^, at 6 h in the SK-MEL-28 melanoma tumour-bearing mice, with tumour to blood ratios of 1.22 at this time point. More recently, using FaDu head and neck cancer xenograft-bearing mice, Tijink *et al* (2006), have also demonstrated maximum tumour localisation of ^125^I-L19-SIP antibody at 6 h post-injection.

When targeting antibodies to solid tumours, factors such as impaired microcirculation in newly formed vessels should be considered, as this may prevent antibody from rapidly reaching its target. Furthermore, the process of crossing the endothelial layer, which precedes the L19 antibody binding to the ED-B antigen on the abluminal surface of the vessel, could be retarded because of interstitial pressure. In addition, the endothelium itself has long been recognised as a natural barrier that may limit the uptake of antibodies in solid tumours. However, in the current study, we did not encounter these problems in our tumour models as both the ^125^I-L19-SIP and Cy3-labelled L19-SIP antibodies (data not shown) had clearly localised to their targets by 1 h after injection. Interestingly, we have observed blood vessel fenestrations in the SW1222 subcutaneous tumours using transmission electron microscopy (data not shown), which may play a role in the extravasation of the L19-SIP antibody and binding to its target, although the involvement of transcytosis cannot be ruled out. These findings, therefore, indicate few barriers to efficient localisation of the L19-SIP antibody in our tumour model; an essential point to be considered when planning timing of future combined therapy regimes.

In the current study, biodistribution experiments demonstrated higher recorded values for tumour localisation of the ^125^I-L19-SIP antibody in the SW1222 xenografts in comparison to LS174T tumours (Figures 2 and 3). Good accessibility and prolonged retention of the Cy3-L19-SIP antibody to its vessel-related target, which was observed as late as 24 h post-injection, was also shown in this tumour model. In view of this prolonged retention, and as ^131^I has a range of emission of approximately 0.8 mm and the thickness of the tumour cord surrounding each tumour vessel in the SW1222 tumours is around 0.5 mm, this isotope was considered the most suitable for RIT using this antibody. Therefore the therapeutic effect of the ^131^I-L19-SIP antibody was subsequently investigated in this model.

Biodistribution experiments, performed to confirm localisation of the therapeutic ^131^I-L19-SIP, showed maximum tumour localisation (8 %ID g^−1^) at 24 h post-injection (Figure 5) in comparison to 6 h for ^125^I-L19-SIP (Figure 2). Interestingly, Tijink *et al* (2006) also demonstrated maximum localisation of ^131^I-L19-SIP (8.6 %ID g^−1^) in FaDu head and neck tumours at 24 h. Although higher radiolabelled antibody levels were found in blood at 3 h in the SW1222 tumour model, this fell dramatically by 24 and 72 h post-injection, indicating good blood clearance and produced high tumour to normal tissue ratios. Indeed, at 24 h, tumour to blood ratio was 28 : 1 (Table 2). This ratio was reduced at 72 h, but still remained high at 9.4. In another study comparing the biodistribution of ^125^I-L19-SIP and ^111^In-L19-SIP in mice bearing the F9 teratocarcinoma tumours, Berndorff *et al* (2005) found that although tumour uptake was equal with both radionuclides, tumour to non-tumour ratios were higher for the ^125^I-labelled antibody.

RIT with ^131^I-L19-SIP caused significant tumour growth delay and improved survival in the SW1222 xenograft-bearing mice. A single dose of 55.5 MBq of radiolabelled antibody significantly inhibited tumour growth for approximately 14 days (Figure 6).

After this time, the majority of the tumours recommenced growth; although one tumour currently appears cured (193 days post-treatment). However, as the group size of the treated mice was small, this finding must be considered as preliminary and would need to be further confirmed in a larger group of mice.

We have shown previously that the blood vessels distribution in SW1222 tumours compose approximately 8% of total tumour mass (El-Emir *et al*, 2005). It is therefore encouraging that, although we are only targeting this relatively small percentage of the total tumour, the amount of localised radiolabelled antibody found by ourselves and other authors is sufficient to produce a significant therapeutic effect. Therefore, it can be considered very promising for clinical trials, and future work will involve demonstrating microscopically whether damage to both endothelium and tumour cells occurs following RIT with ^131^I-L19-SIP.

No significant weight loss was observed in the treated mice indicating that the relatively high doses of radiation used were not toxic. Interestingly, the rapid blood clearance leading to high tumour to blood ratios obtained at the later time points (24 and 72 h) with ^131^I-L19-SIP may have contributed to the absence of toxicity. It is important to note that, in this study, significant results were obtained with doses lower than that used in other RIT studies with the L19-SIP antibody. Berndorff *et al* (2005) reported that a single injection of 74 MBq ^131^I-L19-SIP to F9 tumour-bearing mice resulted in a 10-day tumour growth delay and a median survival of 22 days; 13 days longer than that of control mice. More recently, Tijink *et al* (2006) also demonstrated that a single dose of 74 MBq ^131^I-L19-SIP caused a maximum reduction in FaDu tumour size at day 18. However, at day 26, these tumours had re-grown to their initial size. The results reported in the current study, therefore, compare very favourably with these high-dose therapies, while using 25% less radioactivity (55.5 *vs* 74 MBq), and may be related to the high vascularity of the SW1222 tumours.

In contrast to RIT using antibodies against tumour cell-associated antigens which are expressed only in certain cancers, such as carcinoembryonic antigen in colorectal tumours (Pedley *et al*, 2001; Mayer *et al*, 2003), RIT with L19-SIP antibody, which selectively targets tumour vessels, has the advantage of being effective in a wide range of solid tumours (Berndorff *et al*, 2005; Spaeth *et al*, 2006; Tijink *et al*, 2006). Several options can now be explored to optimise the efficacy of RIT with ^131^I-L19-SIP, for example, repeated injection of the antibody. However, as the L19-SIP is targeting the tumour vessels, which compose only a small percentage of the tumour mass, it is unlikely that the therapy will be totally effective as a single agent. Future experiments will, therefore, concentrate on selecting the optimal combined complementary therapies to overcome the problems of tumour heterogeneity and treat the entire tumour. These combined therapies are most effective when targeting different regions of the tumour. For example, it could be combined with other forms of RIT that target the tumour cells rather than the vasculature (Pedley *et al*, 2001). Tijink *et al* (2006) have shown that the combination of ^131^I-L19-SIP with Cetuximab, an anti-EGFR antibody, enhanced the efficacy of the RIT without causing an increase in toxicity.

In conclusion, this study demonstrates for the first time that ^125^I-L19-SIP and Cy3-L19-SIP showed good tumour selectivity in two human colorectal xenografts with highly different morphologies, and that RIT with ^131^I-L19-SIP caused a significant tumour growth delay and improved survival. This in turn, provides the basis for future combined therapies with RIT.

## Figures and Tables

**Table 1:** Tumour to tissue ratios of ^125^I-L19-SIP distribution in SW1222 and LS174T xenografts-bearing mice

	**1 h**		**3 h**		**6 h**		**24 h**	
**Tumour to tissue ratio**	**SW1222**	**LS174T**	**SW1222**	**LS174T**	**SW1222**	**LS174T**	**SW1222**	**LS174T**
Blood	0.35	0.18	0.83	0.72	1.28	1.28	4.22	3.37
Liver	1.24	0.73	3.31	2.92	4.67	1.78	15.20	8.38
Spleen	1.06	0.46	2.02	2.17	2.86	2.57	7.42	6.12
Kidney	0.38	0.28	1.14	1.15	1.73	1.28	5.03	3.57
Lung	0.85	0.44	1.60	1.37	1.62	1.25	3.53	2.88
Brain	10.93	6.42	31.09	25.32	35.80	29.73	103.72	69.36
Muscle	5.76	4.59	8.45	6.54	8.34	5.84	14.30	12.20
Stomach	1.67	1.47	2.37	1.24	1.14	1.764	2.20	1.60

**Table 2:** Ratios of ^131^I-L19-SIP distribution in SW1222 xenograft-bearing mice

**Ratio**	**3 h**	**24 h**	**72 h**
Blood	0.58	28.11	9.36
Liver	2.01	24.10	33.21
Spleen	0.93	13.47	12.72
Kidney	1.14	7.82	7.62
Lung	1.07	6.95	7.62
Brain	9.71	69.99	135.68
Muscle	3.44	29.39	18.08
Stomach	2.15	4.56	3.60
Colon	4.61	4.62	3.44
